# Study on Anatomical Structures of the Dromedary Udder

**DOI:** 10.3390/ani14111674

**Published:** 2024-06-04

**Authors:** Annika Müller, Ulrich Wernery, Joerg Kinne, Péter Nagy, Judit Juhász, Andre Appelt, Thomas Wittek

**Affiliations:** 1 Vetmeduni Vienna, University Clinic for Ruminants, Veterinärplatz 1, 1210 Wien, Austria; 2 Central Veterinary Research Laboratory (CVRL), Dubai P.O. Box 597, United Arab Emirates; 3 Emirates Industry for Camel Milk Products, Dubai P.O. Box 294236, United Arab Emirates

**Keywords:** dromedary, udder, anatomy

## Abstract

**Simple Summary:**

The objectives of the present study were to describe specific anatomical structures of the dromedary udder. In this study, udders from 86 dromedaries were examined by taking morphological measurements and producing injection casts using resin, gelatin, and paraffin. We examined the different udder, teat, and teat tip shape. The results of this study increase the knowledge of the anatomical structures of the dromedary udder, which may be useful for breeding a selection of dairy dromedaries.

**Abstract:**

The objectives of the present study were to describe specific anatomical structures of the dromedary udder. Eighty-six dromedary udders were examined, taking morphological measurements and producing injection casts using resin, gelatin, and paraffin. The udder of the dromedaries consists of four quarters. The shape of the udders and teats varies considerably between animals and is influenced by age, breeding, and lactation status. The most frequently found udder form was the globular udder (48.8%) and the most common teat form in this study was the funnel teat (44.2%). The most common teat tip shape was a smooth or a slightly rough ring teat (61.6%). Injection casts showed a complete separation of the teat canals. There is also no communication between tributary mammary complexes. Resin injections of the glandular tissue adjacent to the teat cistern showed an extensive branching into large, medium, and small milk ducts. Frozen sections of the udder revealed complete separation of the right and left mammary complex through the *Sulcus intermammarius*. The teat sections showed longitudinal folds from the tip of the teat to the base of the teat. A ring fold at the transition from the *Ductus papillaris* to the teat cistern was present. The results of this study increase the knowledge of the anatomical structures of the dromedary udder, which may be useful for breeding a selection of dairy dromedaries.

## 1. Introduction

Dromedary milk is gaining more and more interest, and camel milk production is increasing. In many countries, like the UAE, Saudi Arabia, and Egypt, the dromedary is now an important source of meat and milk. The animals are no longer only mated without considering breeding success; meanwhile, breeding programs have been established with the aim of increasing milk yield and improving the conformation of the udders. Physiological function and morphology of the mammary gland is important for the production of high-quality milk. However, until now, udder morphology and anatomical structure of the dromedary udder have received little attention. There are only a few studies on the association between udder morphology and milk yield [1,2,3]. The selection of an ideal udder and teat shape is an important step towards adaption to machine milking and helps to develop milking clusters suitable for the machine milking of dromedary camels [4,5]. The mammary gland of the camel consists of four quarters and four teats, like cows [6]. In contrast, the teats have typically two or sometimes three teat canals, and rarely just one [7,8,9]. Each teat canal is associated with one separate tributary glandular complex. The glandular cistern is not a cavity like in cattle but rather resembles a sponge-like structure [8,9,10]. Consequently, the camel udder typically consists of eight separate, independent, milk-producing units [8,11]. Fibroelastic tissue extending from the *Linea alba* and prepubic tendon separates the left and right halves of the udder [12]. The right and left halves of the udder can be differentiated by visual appraisal, but it is not possible to separate the cranial and caudal quarters macroscopically [11,12]. High-performing dairy camels are supposed to have a symmetrically shaped udder with evenly formed teats and a prominent udder vein [13]. However, udder conformation varies depending on different regional types, age, and stage of lactation [8]. The objectives of the study were to determine and describe specific anatomical structures of the dromedary udder using different examination techniques. We hypothesize that the results of this study increase the anatomical knowledge and understanding of the specific anatomy of the dromedary mammary glands, which will support breeding programs and further development of milking machinery for dromedary camels.

## 2. Materials and Methods

### 2.1. Animals

The study was performed on a total of 86 udders. The udders were obtained either from dead or euthanized animals or from slaughtered animals. Additionally, mammary glands were studied in live lactating animals. The udders were removed from 26 female adult dromedary camels submitted for necropsy to the Central Veterinary Research Laboratory (CVRL) in Dubai, UAE. These animals were between 3 and 20 years old. The exact age was unknown due to the lack of documentation from the owners. All these dromedaries died or were euthanized due to diseases that were not associated with the udder. They were in different lactation stages or non-lactating. The number of parities of the adult female dromedary was also different (between 0 and 2 lactations). These udders were used for macroscopic evaluation. Forty-nine udders of female dromedary camels that had been slaughtered in a local abattoir in Al Ain, UAE, were removed immediately after slaughter and transported to CVRL in foam polystyrene boxes on ice. After arriving at CVRL, the udders were macroscopically examined and marked with individual numbers and stored in the cold room (4 °C) until further examination. Unfortunately, no additional data of these animals were available since the farmers were not able to provide information. These udders were used for resin, gelatin, and paraffin casts, measurements, and teat sections to examine the inner structure of the teats. Eleven female dromedary camels in early lactation were randomly selected for measurement in live animals. These animals were owned by the Camelicious (Emirates industry for camel milk products) farm in Dubai, UAE, and were between 14 and 21 years old, with an average of 4.2 lactations.

### 2.2. Udder Measurements and Visual Assessment

**Figure 1:**
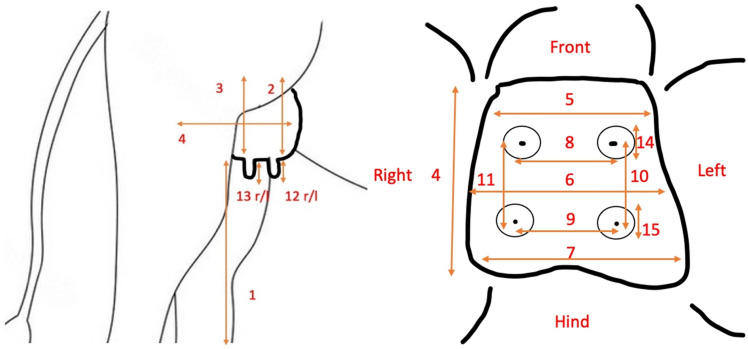
(**Left**) Lateral view on the measurements taken in standing female dromedary camels. (**Right**) View on the measurement taken on the ventral aspect of the camel udders.

Udders of 26 female dromedaries that were delivered for necropsy to the Central Veterinary Research Laboratory and of 11 lactating animals at the Camelicious farm were measured in size, length, and width according to the following procedure (Figure 1, Table 1). Forty-nine udders from the abattoir were also measured using the same procedure, except measurements 1–4 (Figure 1, Table 1) [14,15].

The udder was visually assessed using the following three forms: Pendulous udder, pear-shaped udder, and globular-shaped udder, as described by Ayadi et al. [16] The teat forms were characterized according to the following forms: blow-up teat, cylindrical teat, and funnel teat, as described by AYADI et al. [16]. The teat tip was characterized according to the following forms: no ring teat, smooth or slightly rough ring teat, rough ring teat, and very rough ring teat. Finally, the teats were cut off at the base of the teat and the teat cisterns were measured.

### 2.3. Injection Cast

**Figure 2:**
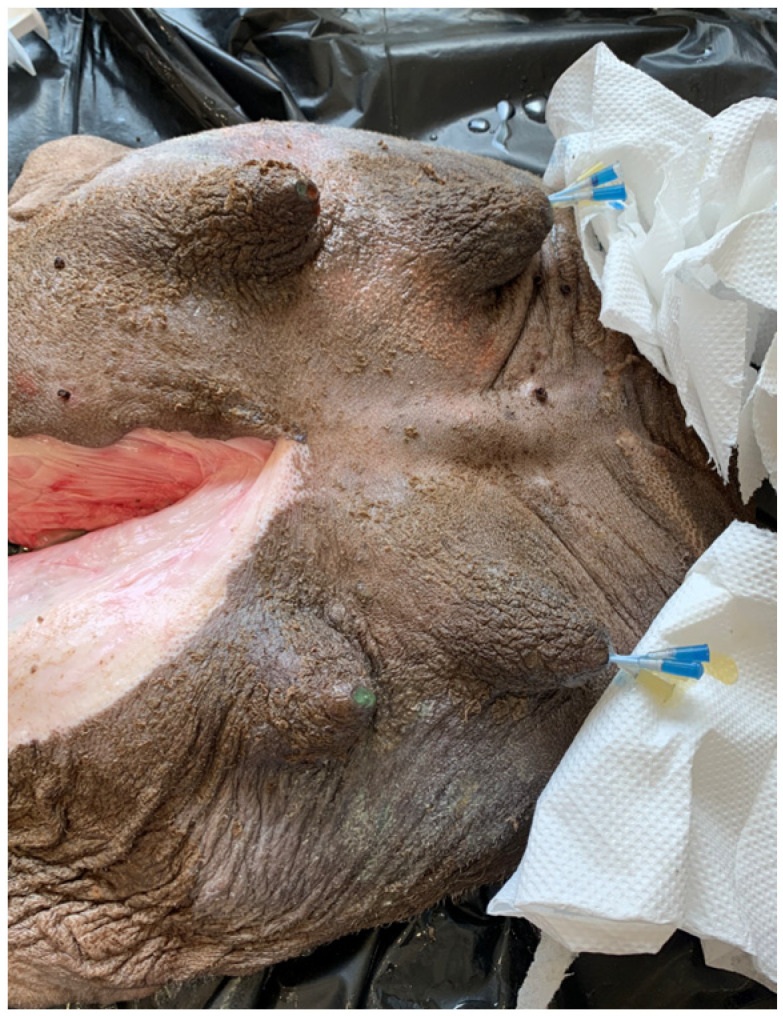
Dromedary udder with catheter placed in the teat canals for resin injection.

Injection casts were made using 10 udders that were randomly selected from the udders obtained from the abattoir. Gelatin, paraffin, or resin solutions were injected (Figure 2) and hardening was allowed. Udders with a resin injection were processed into corrosion casts, while gelatin and paraffin casts were used for macro-freeze sections. During macroscopic examinations of the sections, the following parameters were examined:

- Structure and number of gland complexes per half of the udder;
- Structure and arrangement of the milk ducts;
- Structure of the teat cistern and the teat canal;
- Number of teat canals.

### 2.4. Resin Casts

Resin was injected into six udders. Two catheters (TERUMO SURFLO I.V. Catheter 16 Gx2”, Tokyo, Japan) were inserted into each teat canal. The teat cistern was filled with two-component resin that was colored with oil paint. Two parts EP 150 2–k–Epoxidharz A Component (KLB Kötztal, Ichenhausen, Germany) and one part EP 150 2–k–Epoxidharz B Component (KLB Kötztal, Ichenhausen, Germany) were mixed. The cranial teat canal of the front udder quarter was injected with red-colored resin; for the caudal teat, canal green-colored resin was used. For the rear udder quarters, blue resin was injected into the cranial teat canal and yellow into the caudal teat canal. The resin was injected into the teat canals through the catheter with a 60 mL syringe until the mammary complex was filled. The catheters were left in the teat canals for about 15 min. After a further 30 min, the udder was turned over and hung in the correct anatomical position. Hardening of the resin in this position was allowed for 24 h. After 24 h, the excess tissue was removed with a scalpel and the cast was placed for six hours in boiling water. For maceration, it was soaked in detergent (Papain, 1:20) and incubated (Heraeus Incubator Thermo-Scientific, Darmstadt, Germany) at 60 °C. After three to six weeks, the tissue was completely macerated depending on the size of the udder. The casts were carefully washed under running water and then dried for one week. Photographic pictures were taken by placing the casts on a white background.

### 2.5. Gelatin Casts

A second type of injection cast was made of one udder using commercial bovine gelatin (Davis gelatin, Eberbach, Germany). A mixture of a teaspoon of gelatin was stirred into a cup of water. Green or red food coloring was added to the front teats, and blue or yellow was used for the rear teats as described for the resin casts. If there was an additional third teat canal, pink was used. The gelatin was mixed with cold water and heated in a water bath while stirring constantly with a whisk. A syringe (10 mL) was meanwhile warmed up in warm water. The udder was placed on a flat surface and an indwelling venous catheter (TERUMO SURFLO I.V. Catheter 16 Gx2”, Tokyo, Japan) was inserted into each *Ostium papillare*. The gelatin solution (two times 10 mL) was injected into the individual teat canals with the warmed-up syringe. After the injection, the indwelling vein catheter was removed and the *Ostium papillare* was kept closed with a fingertip while applying slight pressure until the gelatin had hardened to such an extent that it no longer leaked. After 45 min, the udder was placed in physiological position and frozen at −20 °C. The frozen udder was cut transversally in several planes using a band saw (Kolbe Foodtec K220, Elchingen, Germany) and the cut surfaces were photographed with a digital camera. The sections were then fixed in formalin for two weeks and then placed in PEG 4000 (Bauer Handels GmbH, Fehraltorf, Switzerland) for a further two weeks.

### 2.6. Paraffin Casts

The third type of injection cast was made using paraffin (Thermoscientific Histoplast PE, Schwerte, Germany), which was melted in a water bath. For coloring, an oil paint was added; red and green was used for the front teat canals and yellow and blue for the rear teats as described for the resin casts. An indwelling vein catheter (TERUMO SURFLO I.V. Catheter 16 Gx2”, Tokyo, Japan) was introduced into each *Ostium papillare* and the paraffin was injected using a syringe (10 mL). Depending on the size of the mammary complex, between 1 and 20 mL of paraffin were injected. The injection was stopped when the increasing pressure prevented further injection. After the injection, the indwelling vein catheter was removed and the *Ostium papillare* was closed with the fingertip until the paraffin had hardened (approx. two minutes). After 45 min, the udder was placed in physiological position at −20 °C until it was completely frozen. The frozen udder was cut transversally and longitudinally in several planes using a band saw (Kolbe Foodtec K220, Elchingen, Germany) and the cut surfaces were photographed with a digital camera. The sections were then fixed in formalin for two weeks and then placed in PEG 4000 (Bauer Handels GmbH, Fehraltorf, Switzerland) for a further two weeks or individually wrapped and stored in the freezer.

### 2.7. Frozen Section

Native frozen sections were prepared from two udders. These udders were frozen in physiological position with the help of narrow wooden boards at −20 °C. The frozen udders were cut transversally and longitudinally in several planes using a band saw (Kolbe Foodtec K220, Elchingen, Germany) and the cut surfaces were photographed with a digital camera. The sections were fixed in formalin for 2 weeks and then placed in PEG 4000 (Bauer Handels GmbH, Fehraltorf, Switzerland) for a further 2 weeks or individually wrapped and stored in the freezer.

### 2.8. Teat Sections

Teat sections were made from eight udders. To do so, 32 teats were separated at the base and then each teat canal was individually cut open with scissors. The various sections were recorded with a digital camera.

### 2.9. Statistical Analysis

The statistical evaluation was carried out using Excel Version 16.78. Data were checked for normal distribution and then the median, first, and third quartiles were calculated. The data were log transformed and the one-way ANOVA test was used to compare the measurements taken from udders from necropsy, abattoir, or lactating animals. To examine the measured values of the individual groups for significant differences, the Bonferroni post hoc test was applied. A *p* < 0.05 was considered to indicate statistical significance.

## 3. Results

### 3.1. Macroscopic Examination

The udder of the dromedary is suspended between the hind legs. It consists of two halves with two teats each. Both halves are separated from each other by a longitudinal structure called *Sulcus intermammarius*. The shape of the udder and teat varies with age, breeding, and lactation status. For most udders, the front teats are slightly wider apart than the rear teats (Table 2). The skin of the udder and teat is rough, of a brownish grey color. There are no hairs on the teat and only short, fine hair at the teat base. The udder is usually completely hairy. The color of the hair varies from beige to dark brown or brown-grey. Most teats had two teat canals (329 teats/95.6%) that were completely independent of each other and associated with a separate glandular complex. Rarely did only one teat canal (2 teats/0.6%) or three teat canals (13 teats/3.7%) per teat occur.

### 3.2. Measurements

Table 2 gives an overview of the measurements of the camel udders.

### 3.3. Udder Forms

**Figure 3:**
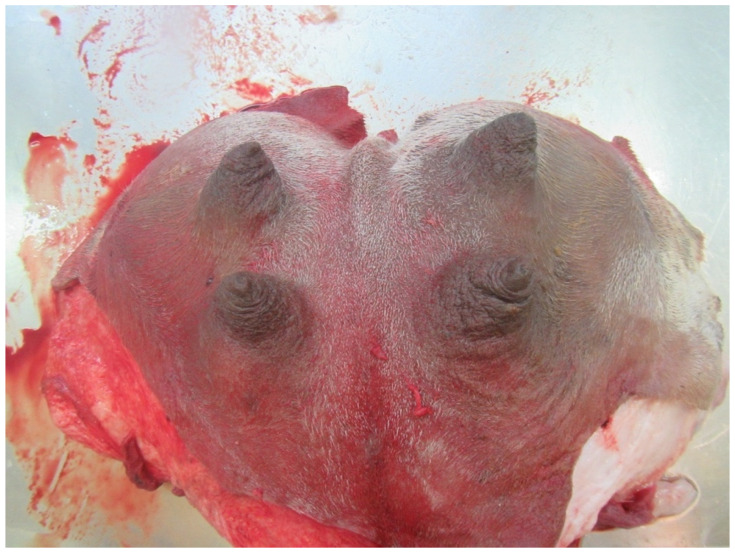
Globular udder with funnel teats.

**Figure 4:**
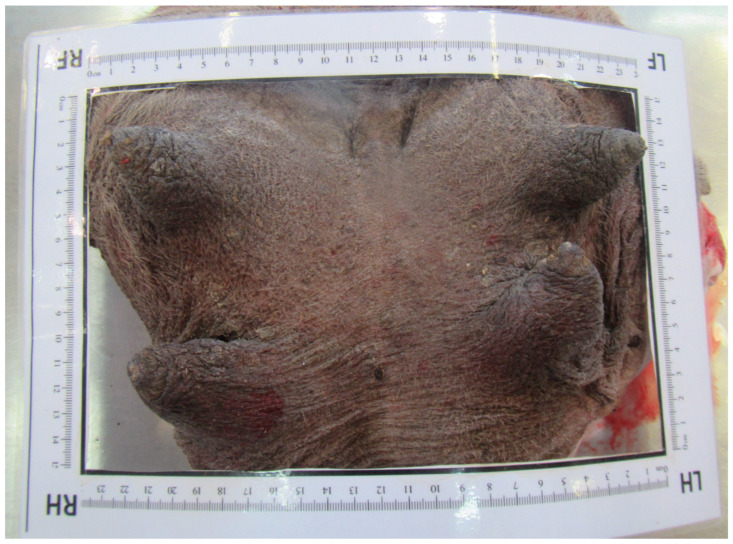
Pear-shaped udder with cylindrical teats.

**Figure 5:**
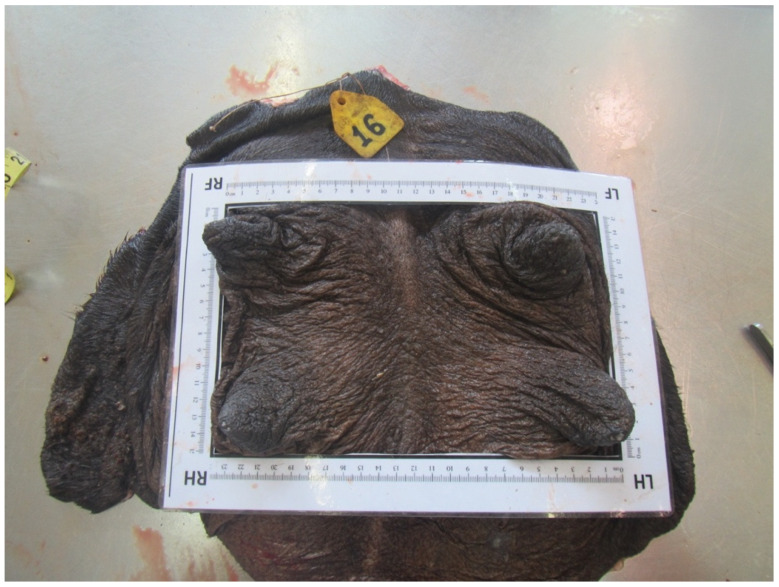
Pendulous udder with blown-up teats.

Three different udder shapes occurred: pendulous udder, pear udder, or globular udder, shown in Figure 3, Figure 4 and Figure 5. Table 3 gives an overview of the distribution of the udder forms.

### 3.4. Teat Forms

Three types of teats (blown-up teat, cylindrical teat, and funnel teat) were differentiated. Table 4 gives an overview of the distribution of the teat forms (Figure 3, Figure 4 and Figure 5).

### 3.5. Teat Tip Shape

**Figure 6:**
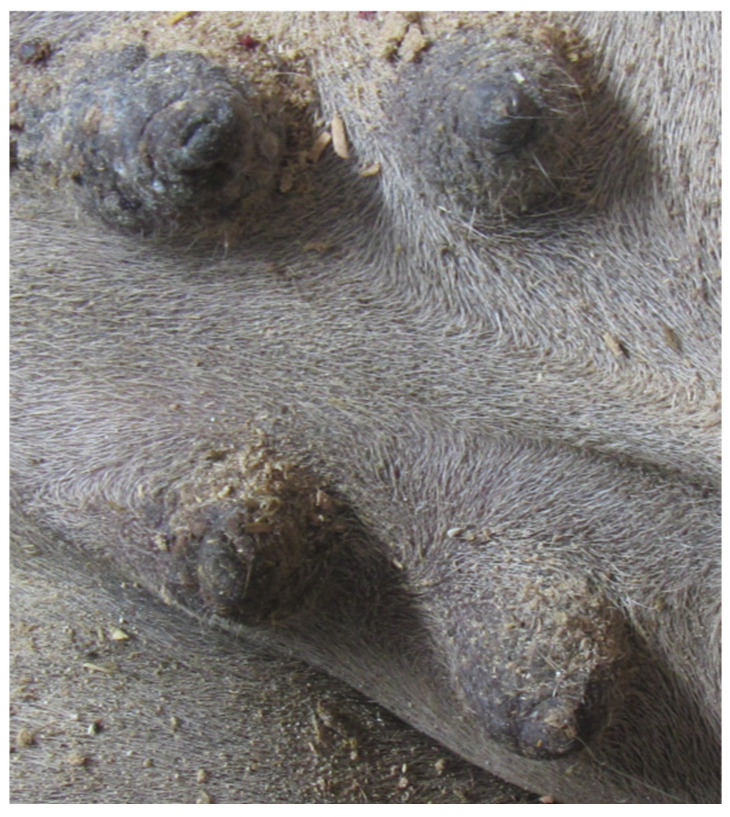
Juvenil udder with no ring teat.

**Figure 7:**
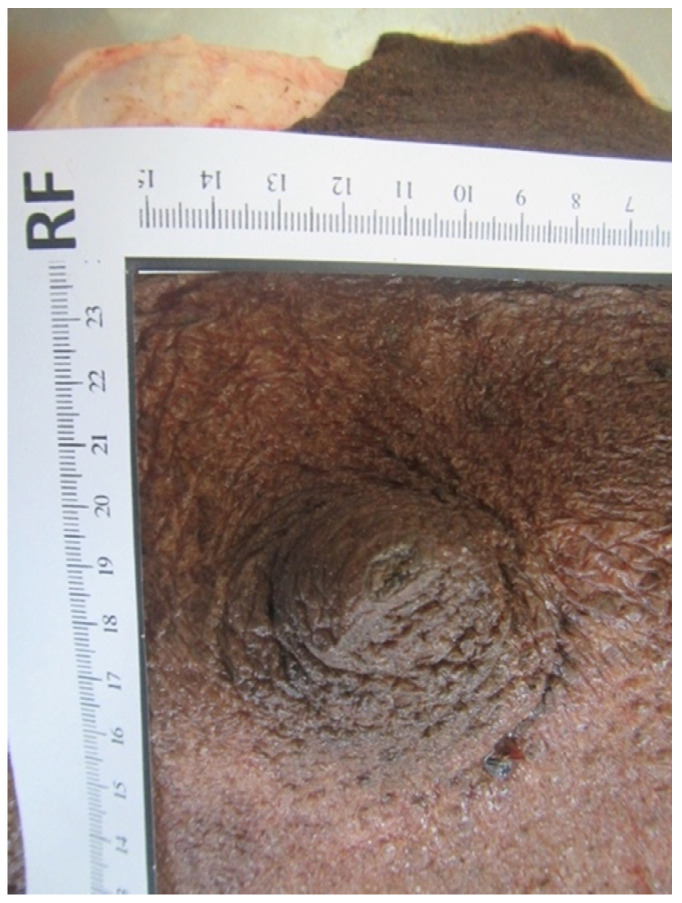
Right front teat with a smooth or slightly rough ring teat.

**Figure 8:**
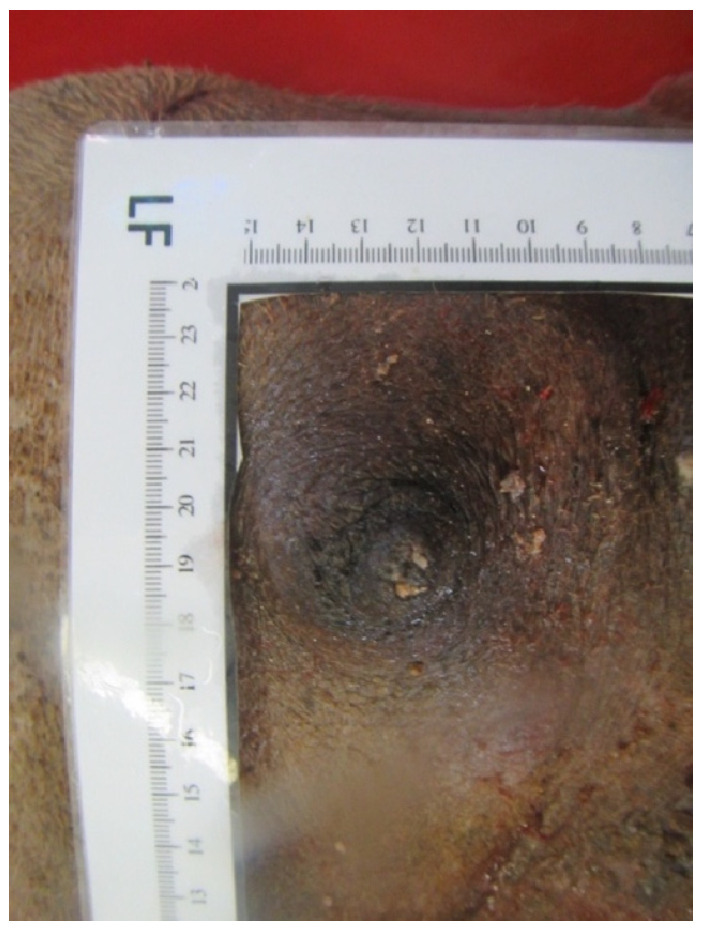
Left front teat with a rough ring teat.

**Figure 9:**
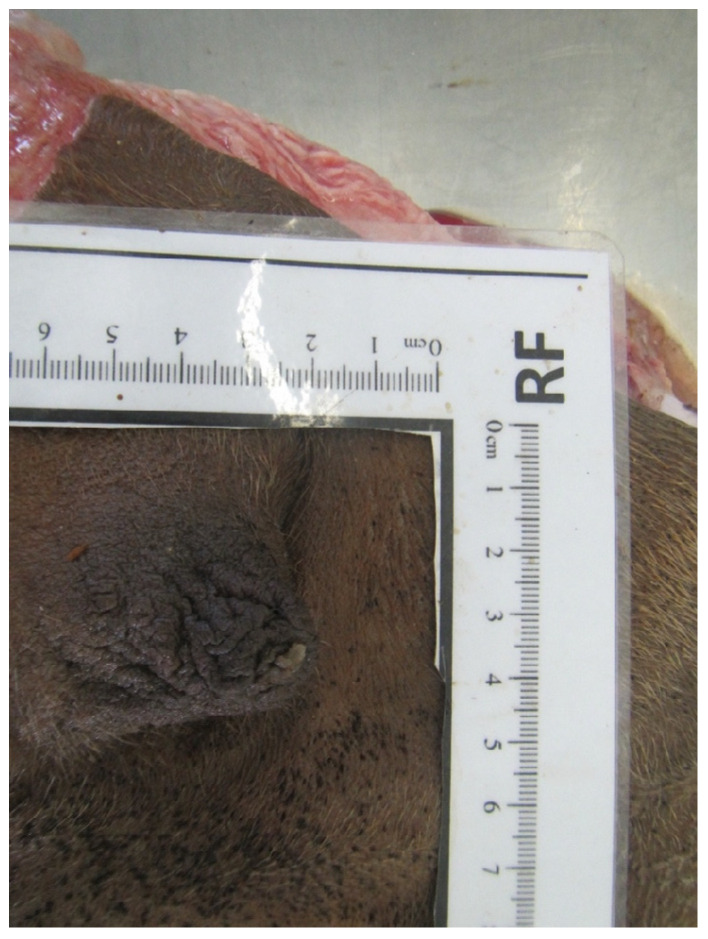
Right front teat with a very rough ring teat.

Four different shapes of the tip of the teat were defined: no ring teat, smooth or slightly rough ring teat, rough ring teat, and very rough ring teat. Table 5 gives an overview of the distribution of the teat tip shape (Figure 6, Figure 7, Figure 8 and Figure 9).

### 3.6. Paraffin Injection

**Figure 10:**
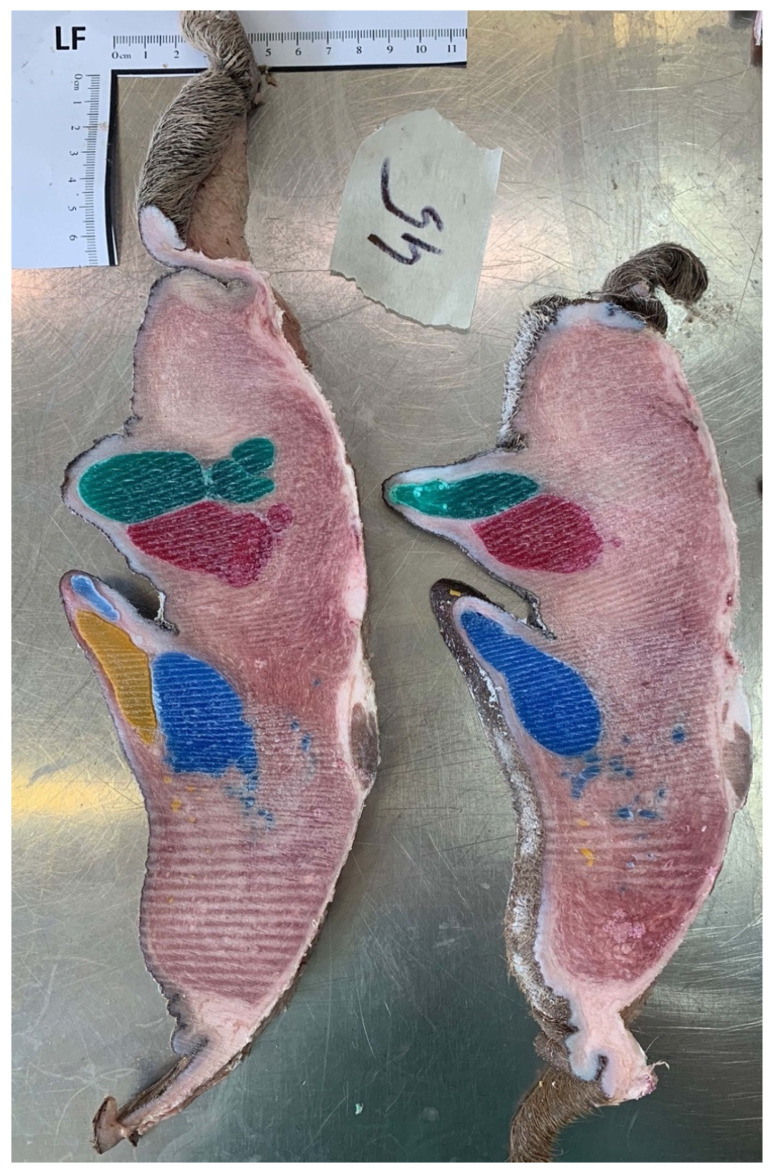
Injection cast with colored paraffin (red and green for front teats, yellow and blue for rear teats). Frozen section, two teat canals in each teat on the left side.

In all three paraffin-injected udders, two *Ostium papillare* existed in each teat. In each teat canal, 20 mL of paraffin were applied. A clear separation of the two teat canals per teat was seen (Figure 10). Folds were visible at the transition from the *Ductus papillaris* to the teat cistern in all teats. In addition to the formation of wrinkles, shallow depressions were noticed on the inner wall of the teat cistern. These depressions led into the fine canals of the glandular tissue. It was not possible to inject the paraffin into the narrower glandular ducts.

### 3.7. Gelatin Injection

**Figure 11:**
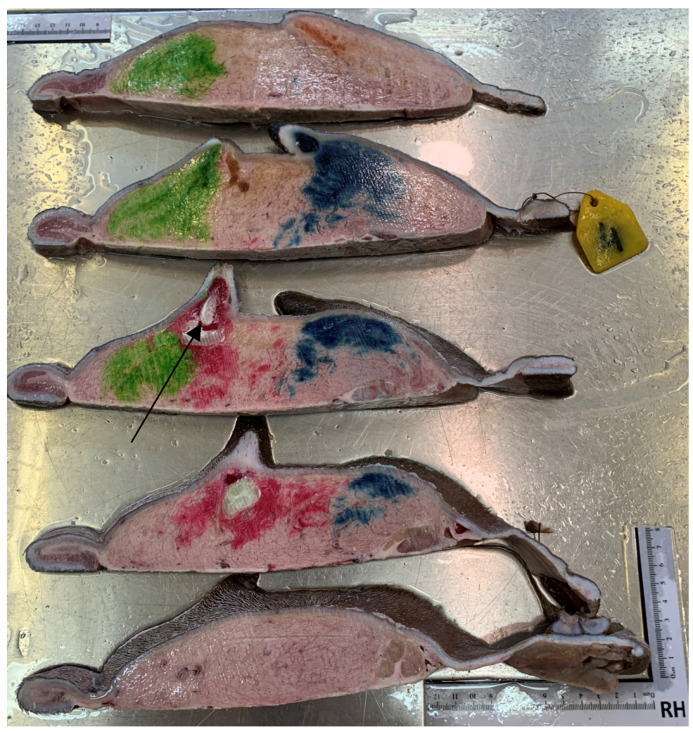
Injection cast with colored gelatin (red and green for front teats, yellow and blue for rear teats). Frozen section, two teat canals in each teat of the right side and clear separation of the parenchyma (arrow).

One udder from the abattoir was randomly selected for gelatin injection. This udder had two *Ostium papillare* in each teat and 20 mL of gelatin were injected into each teat canal. The entire gland tissue was colored in the respective color of the associated teat canal (Figure 11). However, individual ducts could not be identified. Depending on the how the filling of the teat cistern was, folds could be seen mainly in the proximal area of the teat.

### 3.8. Resin Injection

**Figure 12:**
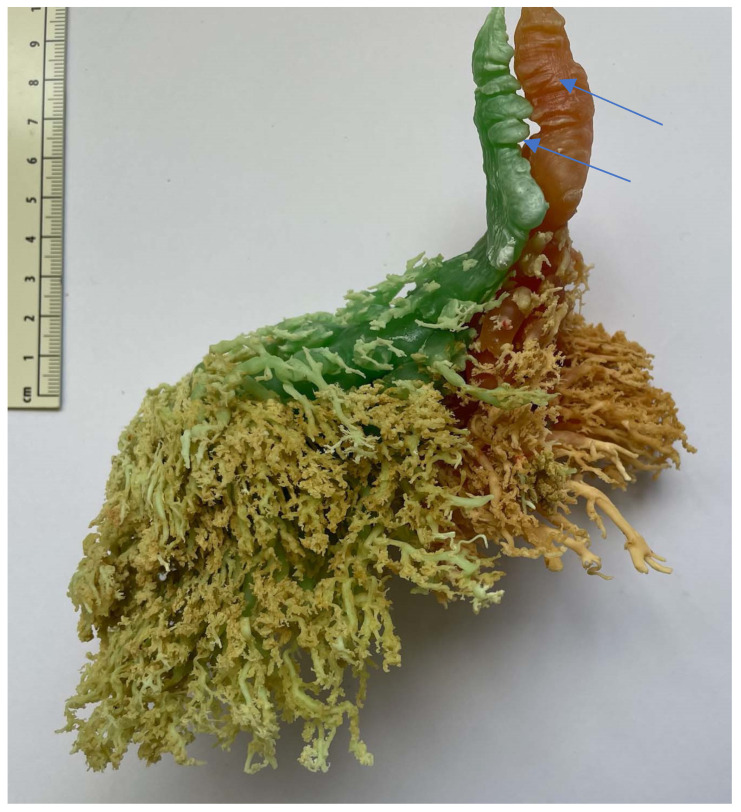
Injection cast with colored resin. Two complexes (green and red) of a front teat in lateral view. The arrows mark the fold-like, ring-shaped constrictions in the teat cistern.

**Figure 13:**
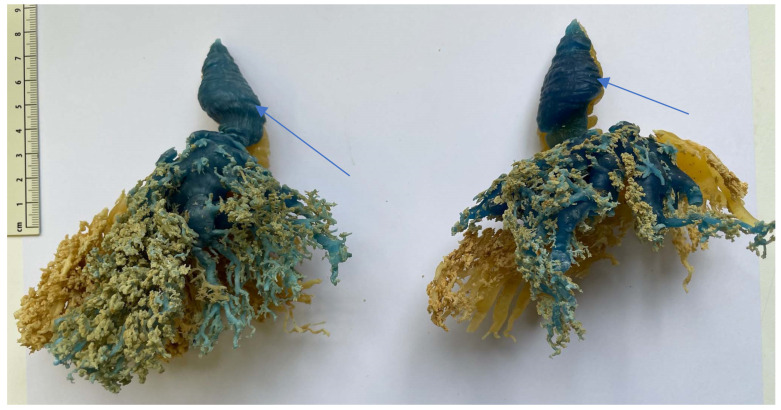
Injection cast with resin. Two complexes (blue and yellow) of a hind teat in lateral view. The arrows mark the fold-like, ring-shaped constrictions in the teat cistern.

Six udders were used for with resin injections. Between 21 mL and 119 mL of differently colored resin were injected in each canal (mean 53 mL). No connection between the complexes or the teat cisterns was present (Figure 12 and Figure 13). In the teat cistern, fold-like constrictions were found along the entire lumen wall. These folds were ring-shaped in the horizontal position. At the transition to the *Ductus papillaris* from the teat cistern, several small, wrinkle-like indentations were visible. Large, medium, and small milk ducts could be visualized in the glandular tissue (Figure 13). When examining the individual udders, no uniform principle of the milk ducts could be determined in terms of width and shape. Following the teat cistern, several larger milk ducts followed, which branched out into many small, fine ducts and formed a spongy pattern.

### 3.9. Frozen Sections

**Figure 14:**
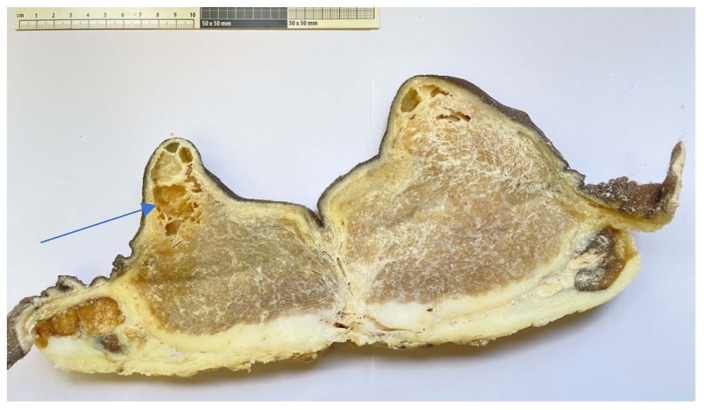
Native frozen section fixed in formalin. The arrow marks the fold-like, ring-shaped constrictions in the teat cistern.

Two udders from the abattoir were used for native frozen sections. These frozen sections gave a closer look at the structural arrangement of the dromedary udder. A clear separation of the right and left mammary complex through the *Sulcus intermammarius* can be seen. Fine ducts stand out in the glandular tissue through the frozen milk. The lumen of the teat cistern is lined with fold-like, ring-shaped constrictions (Figure 14). The folds show a heterogeneous appearance in terms of their size and shape.

### 3.10. Teat Sections

**Figure 15:**
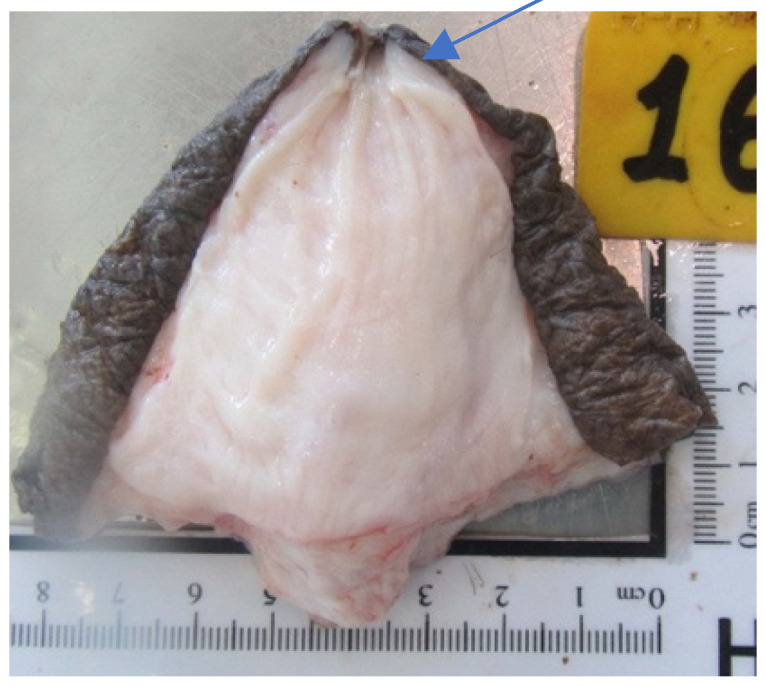
Cutting plane of a teat canal and teat cistern after separating the teat at the base from the udder. The arrow marks the ring fold at the transition from the *Ductus papillaris* to the teat cistern.

**Figure 16:**
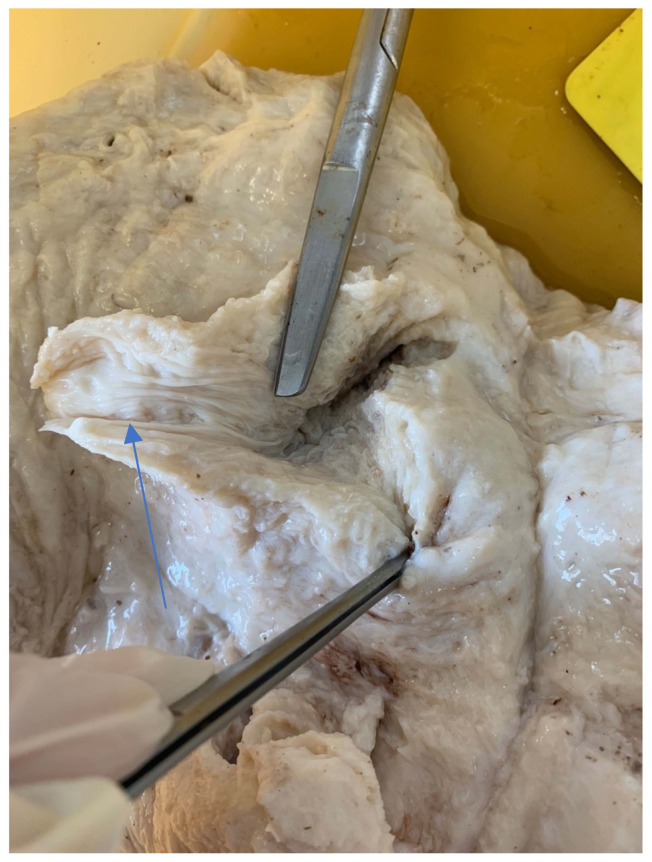
Sectional plane of a teat canal and teat cistern on an udder fixed in formalin. The longitudinal folds (arrow) are visible.

Teat sections were made from eight udders from the abattoir. Several longitudinal folds protruded from the wall into the lumen of the teat cistern (Figure 15 and Figure 16). These folds ran from the tip of the teat to the base of the teat. A ring fold was visible at the transition from the *Ductus papillaris* to the teat cistern (arrow, Figure 15). Several longitudinal folds can be seen in the proximal section of the teat cistern.

## 4. Discussion

### Measurements of the Udder and the Teats

Eisa et al. [17] (16 dromedaries) and Musaad et al. [18] (72 dromedaries) described that lactating dromedaries have a large variation in morphology and the size of the teats and udders. Our examination of udders from three different sources showed very similar results for the individual measured values, with significant differences comparing the udders from necropsy and abattoir with the udders of the live animals. To what extent this is due to breeding could not be determined, as there was no information of origin for most animals. However, most likely, all these camels came from the same region. The diameter in the middle of the teat was about 3.7 cm for the necropsy and abattoir udders. This is larger than those reported for Saudi Arabian lactating camels managed in comparable conditions [1] but similar to the results of [15]. The udders of the living animals had particularly large teat diameters (about 6 cm), most likely due to adaptation to machine milking. Atigui et al. [15] stated that multiparous camels had significantly larger teats and bigger udders than primiparous camels. This could be related to the lactation state and the age. However, there was a significant difference between living and dead animals in our examinations.

The results of different examinations and measurements showed a variability in the individual udders. Lactating dromedaries had a large variation in morphology and size of teats and udders [1,6,17]. On one hand, this is related to the daily milk yield. Nagy and Juhasz [19] described that, in general, a larger udder also has a greater potential for a higher milk production. Musaad et al. [18] stated that pear-shaped udders produced significantly more milk than pendulous udders. Another issue is that systematic breeding in dairy camels is only slowly starting. Different shape and size for teats is a constant problem for the correct position of the milking clusters by machine milking. Incorrect position can lead to mastitis.

The most common form of udders in live animals is pear udders (54.5%). However, the most common form of abattoir udders and animals delivered for necropsy was the globular udder (abattoir: 57.1%, necropsy: 38.4%). To what extent this is related to the state of lactation could not be clarified, since there was no documentation on the udders from the abattoir. There were also no data available on the lactation stage or milk yield for most of the animals that were submitted for necropsy. Ayadi et al. [20] reported that globular-shaped udders were the most common (47.3%) in Arabian dairy camels, followed by pear (34.3%) and pendulous (18.4%) shapes. This was almost identical to our observation (globular: 48.8%, pear: 27.9%, pendulous: 17.4%).

Teats also showed wide variability both in shape and in size. In the present work, three teat forms, cylindrical, funnel, and blown-up teats, were observed. This is similar to what had been described in dairy cattle [21] as well as for dromedaries [18]. Funnel teats were the most frequent shape (44.2%), followed by cylindrical (36.0%) and blown-up teats (17.4%). To clearly describe the different shapes, a more detailed differentiation of the teats into five different categories as used by Nagy et al. [2] would be useful. They describe cylindrical, conical, conical with base, conical–cylindrical, and deformed teats. To further describe the teat unequivocally, it would be better to measure not only the diameter in the middle of the teat but also at the tip and at the base. The mean teat length of the lactating animals was longer than of the abattoir udders and the animals delivered for necropsy (living: 6 cm, abattoir: 4.6 cm, necropsy: 5.3 cm). There was a significant difference between the udders of the lactating camels and the udders of the abattoir. Nagy and Juhasz [19] found that the animals from the Camelicious farm have longer teats (mean 7.1 cm) than other authors did, like Eisa et al. [17] (mean length 4.3 to 4.4 cm) and Ayadi et al. [1] (mean length 4.3 to 5.3 cm), who also analyzed living lactating camels, most likely related to different breeds. An advantage of longer teats can be a better fit of the milking clusters and easier milking by hand. The disadvantage of longer teats is that they come close to the ground when lying down and making it easier for infections to occur via the litter or sand. Before milk ejection and during milking, teats of dromedaries go through tremendous changes [19].

The extent to which the different forms lead to the development of mastitis must be further clarified by more examination. Seykora and McDaniel [22] stated that firmly attached, higher udders are less susceptible to mastitis than pendulous udders in bovine. Sinha et al. [23] described that bovines with broader udder width, weaker fore udder attachment, and longer rear teats seem to be more susceptible to mastitis. Young et al. [24] concluded that deeper bovine udders have higher cell counts and more incidence of mastitis. This could not be clarified in detail in the present work and requires further examination.

Another important structure in preventing mastitis is the teat orifice. In the present work, a distinction was made between five forms: smooth ring teat, no ring teat, smooth or slightly rough ring teat, rough ring teat, and very rough ring teat. Most animals showed a smooth or slightly rough ring teat. It is particularly important that the opening does not show any changes since any change, like hyperkeratosis, increases the risk for bacterial invasion. Hyperkeratosis on the teat tips prevents the teat canal from closing quickly and completely after milking. In consequence, pathogens from the teat skin and from the stable surfaces can enter the udder and may cause mastitis. That is why it is important to avoid overmilking with blind milking phases and to install milking clusters suitable for dromedary camels [14].

The ring fold formation in the teat cistern is a protective mechanism of the dromedary udder.

When examining the injection casts and the frozen sections (Figure 15), horizontally arranged ring folds could be seen in the teat cisterns. Rizk et al. [11] addressed these folds as the Fürstenberg rosette. The number of folds varied between 10 and 14 in our study, similar to what has been described by Kausar et al. [25], who found 12 to 14 folds in dromedaries from Pakistan. This is similar to what was reported in buffalo by Nickereson [26], ranging from 10 to 14.

In dromedaries, the Fürstenberg rosette does not form a ring at the transition from the teat canal to the teat cistern as in cattle, but the folds reach into the cistern up to about the middle of the teat. One ring fold can also be seen at the transition from the teat canal to the teat cistern. This mechanical barrier makes it difficult for bacteria and germs to penetrate. Another function of the folds may be a more controlled milk release. In our cases, several ring-shaped, wrinkle-like constrictions were noticed in the injection casts (Figure 12) and the frozen sections (Figure 14).

## 5. Conclusions

The injection casts and frozen section of the udder of the dromedaries shows a separation between the two teat canals and the two glandular complexes of each quarter. The structure of the teat and the parenchyma shows that the dromedary has specific anatomical structures that are important to know for the application of machine milking and for improving udder health.

## Figures and Tables

**Table 1:** Description of the measurements taken on the udders of standing (living) or lying (necropsy) animals (Figure 1, left) and on the ventral aspect of the dromedary camels (Figure 1, right) in cm.

1	Height of the udder above ground (the distance between the right hind teat base and the ground)
2	Front udder depth (the distance between the udder attachment at the abdomen and the base of the teats) right front
3	Hind udder depth (the distance between the udder attachment at the abdomen and the base of the teats) right hind
4	Udder length (the distance between the most cranial and the most caudal part of the udder)
5	Front udder width (the distance between the one side to the other before the front teats)
6	Mid udder width (the distance between the one side to the other in the middle between the most cranial and the most caudal part of the udder)
7	Hind udder width (the distance between the one side to the other behind the hind teats)
8	Front teat distance (distance between the front teats, measured from the middle of the teat)
9	Hind teat distance (distance between the hind teats, measured from the middle of the teat)
10	Left teat distance (distance between the left front and left hind teat, measured from the middle of the teat)
11	Right teat distance (distance between the right front and right hind teat, measured from the middle of the teat)
12	Front teat length (distance from the teat insertion base to the teat orifice) right and left front
13	Hind teat length (distance from the teat insertion base to the teat orifice) right and left hind
14	Front teat diameter (measured in the middle of the teat) right and left front
15	Hind teat diameter (measured in the middle of the teat) right and left hind

**Table 2:** Udder measurement of 86 dromedary camels (in cm), with data provided as median, first, and third quartiles.

		Necropsy Udder (26 Udders)	Abattoir Udder (49 Udders)	Living Animal Udder (11 Udders)
		First Quartile/Median/ Third Quartile	First Quartile/Median/ Third Quartile	First Quartile/Median/ Third Quartile
1	Height of the udder over ground	92.7/111.5/121.3		101.0/107.0/109.0
2	Front udder depth	12.1/25.0/30.3		28.0/31.0/32.0
3	Hind udder depth	10.0/15.5/22.2		32.0/34.0/38.0
4	Udder length	21.0/29.2/35.0		46.0/48.0/52.0
5	Front udder width	25.5/32.5/42.0	27.0/31.0/34.0	40.0/49.0/59.0
6	Mid udder width	29.2/39.0/45.5	31.0/35.0/39.5	58.0/59.0/66.0
7	Hind udder width	23.0/32.7/37.4	26.5/29.0/32.5	57.0/65.0/69.0
8	Front teat distance	11.7/16.0/20.2	13.0/16.5/19.0	16.0/19.0/21.0
9	Teat distance	8.5/13.7/18.6	12.5/14.5/16.2	9.0/13.0/14.0
10	Left teat distance	6.3/8.2/11.6	6.5/8.0/10.0	6.5/7.0/8.0
11	Right teat distance	6.5/8.5/11.6	6.5/8.5/10.0	7.0/7.5/8.0
12	Front teat length left	3.8/5.5/6.6	3.5/4.5/5.5	5.0/6.0/7.5
	Front teat length right	3.5/5.6/6.5	3.5/4.5/5.5	5.0/5.0/6.0
13	Hind teat length left	4.2/5.4/6.0	3.5/4.5/5.2	6.0/6.5/8.0
	Hind teat length right	3.4/5.0/5.8	3.8/4.9/5.5	5.0/6.5/7.0
14	Front teat diameter left	2.6/3.7/4.2	2.7/3.4/4.5	4.5/5.5/7.0
	Front teat diameter right	2.2/3.5/4.9	2.9/3.7/4.6	4.0/6.0/6.5
15	Hind teat diameter left	2.5/3.4/5.1	2.8/3.4/3.8	5.5/6.0/7.0
	Hind teat diameter right	2.7/3.5/4.6	2.9/3.4/4.2	5.0/5.0/6.0

**Table 3:** Frequency and distribution of the udder forms of dromedary camels.

	Pendulous Udder	Pear Udder	Globular Udder	Juvenil	Total
Necropsy	5	6	10	5	26
Living animals	1	6	4		11
Abattoir	9	12	28		49
Total number/Percentage	15/17.4%	24/27.9%	42/48.8%	5/5.8%	86

**Table 4:** Frequency and distribution of the teat forms of dromedary camels.

	Blown-Up Teat	Cylindrical Teat	Funnel Teat	Total
Necropsy	3	14	9	26
Living animal	2	6	3	11
Abattoir	10	12	27	49
Total number/Percentage	15/17.4%	32/37.2%	39/45.3%	86

**Table 5:** Frequency and distribution of the teat tip shape of dromedary camels.

	No Ring Teat	Smooth or Slightly Rough Ring Teat	Rough Ring Teat	Very Rough Ring Teat	Total
Necropsy	1	17	6	2	26
Living animal	0	11	0	0	11
Abattoir	0	25	19	5	49
Total number/Percentage	1/1.16%	53/61.6%	25/29.1%	7/8.1%	86

## Data Availability

The datasets generated during the current study are not publicly available.
